# Nutraceutical Potential of *Carica papaya* in Metabolic Syndrome

**DOI:** 10.3390/nu11071608

**Published:** 2019-07-16

**Authors:** Lidiani F. Santana, Aline C. Inada, Bruna Larissa Spontoni do Espirito Santo, Wander F. O. Filiú, Arnildo Pott, Flávio M. Alves, Rita de Cássia A. Guimarães, Karine de Cássia Freitas, Priscila A. Hiane

**Affiliations:** 1 Graduate Program in Health and Development in the Central-West Region of Brazil, Federal University of Mato Grosso do Sul-UFMS, MS 79079-900 Campo Grande, Brazil; 2 Faculdade de Ciências Farmacêuticas, Alimentos e Nutrição, Federal University of Mato Grosso do Sul-UFMS, MS 79079-900 Campo Grande, Brazil; 3 Institute of Biosciences, Federal University of Mato Grosso do Sul-UFMS, MS 79079-900 Campo Grande, Brazil

**Keywords:** blood glucose, food composition, metabolic syndrome, natural products, *Carica papaya*

## Abstract

*Carica papaya* L. is a well-known fruit worldwide, and its highest production occurs in tropical and subtropical regions. The pulp contains vitamins A, C, and E, B complex vitamins, such as pantothenic acid and folate, and minerals, such as magnesium and potassium, as well as food fibers. Phenolic compounds, such as benzyl isothiocyanate, glucosinolates, tocopherols (α and δ), β-cryptoxanthin, β-carotene and carotenoids, are found in the seeds. The oil extracted from the seed principally presents oleic fatty acid followed by palmitic, linoleic and stearic acids, whereas the leaves have high contents of food fibers and polyphenolic compounds, flavonoids, saponins, pro-anthocyanins, tocopherol, and benzyl isothiocyanate. Studies demonstrated that the nutrients present in its composition have beneficial effects on the cardiovascular system, protecting it against cardiovascular illnesses and preventing harm caused by free radicals. It has also been reported that it aids in the treatment of diabetes mellitus and in the reduction of cholesterol levels. Thus, both the pulp and the other parts of the plant (leaves and seeds) present antioxidant, anti-hypertensive, hypoglycemic, and hypolipidemic actions, which, in turn, can contribute to the prevention and treatment of obesity and associated metabolic disorders.

## 1. Introduction

Plants with healing properties are utilized in folk medicine and, since remote times, have been considered traditional therapeutic approaches that have effects on health. They are also advantageous from a cost–benefit point of view [1]. Synthetic drugs used to be the first option for the treatment of several diseases. However, because of the adverse effects shown by long- or even short-term consumption, studies aiming at the use of alternative therapies in the treatment and prevention of diseases have increased considerably [2]. 

One alternative therapy includes the use of nutraceuticals, which, in turn, according to the existing regulations, cannot be categorized or defined either as food or a drug, but can be understood in the category of food supplements, with beneficial properties for health maintenance, in particular for some pathologic conditions. Therefore, a therapeutic approach, based on nutraceuticals for maintenance of health, resulted in a worldwide “nutraceutical revolution” [3]. 

Among plants with beneficial properties on health is *Carica papaya*, the well-known papaya. This fruit contains considerable concentrations of vitamins, bioactive compounds and a lipidic composition that reduces inflammatory markers and anti-platelet aggregation, protects against thrombogenesis and oxidative stress, and prevents hypercholesterolemia—factors that can be triggered by obesity [4,5].

*Carica papaya* is a popular fruit, and its largest production occurs in tropical and subtropical regions. According to the Food and Agriculture Organization of the United Nations (FAO) [6], over 6.8 million tons of the fruit are produced in the world annually, ca. 440 thousand ha. Central and South America, especially Brazil, are responsible for 47% of the fruit yield, produced year round, being an important source of nutrients with a low cost and great availability in the market.

*Carica papaya* is consumed worldwide, either in natura or processed as jam, sweets and pulp, and to aggregate the nutritional value, other parts of the plant (leaves and seeds) are added to some products in the form of teas and flours [7]. The pulp composition presents three important sources of vitamins with potential antioxidant action, A, C and E [8], besides minerals, such as magnesium and potassium, and B complex vitamins, such as pantothenic acid and folate [9], as well as the presence of food fibers [10]. Besides these nutrients, papaya contains the enzyme papain, effective in increasing intestinal motility and transit time, and is also utilized in the treatment of traumas, allergies and sport lesions [5]. Some studies observed the presence of proteolytic enzymes, such as chymopapain, with anti-viral, antifungal and antibacterial properties [5,11].

The seed contains phenolic compounds, such as benzyl isothiocyanate, glucosinolates, tocopherols (α and δ), β-cryptoxanthin, β-carotene and carotenoids [12,13], while the seed oil principally presents oleic fatty acid, followed by palmitic, linoleic and stearic acids [14]. The leaves have a high content of food fibers and polyphenolic compounds, such as flavonoids, saponins, pro-anthocyanins, tocopherol and benzyl isothiocyanate [15].

Considering the nutrients present in its composition, beneficial effects have been observed, with a significant improvement in the cardiovascular system, protecting against cardiovascular illnesses, heart attack and strokes [16]. Other studies have pointed out that this fruit is an excellent source of beta-carotene (888 IU/100 g), preventing harms caused by free radicals [17], besides exerting a role in the prevention of cardiovascular illnesses, diabetes mellitus (types 1 and 2) and in the reduction of cholesterol levels through its high content of fibers, which diminish fat absorption [5,18].

*Carica papaya* is a plant that is easily accessed and widely available. Furthermore, scientific studies have demonstrated the biological activities and medicinal applications of different parts of the plant. However, few studies have demonstrated the therapeutic potential in metabolic dysfunctions in experimental models specific to obesity. Therefore, the present study will investigate the nutritional value and bioactive compounds of the plant, as well as the existing medicinal uses and possible application in the metabolic syndrome.

## 2. Nutritional Properties: *C. papaya* L.

### 2.1. Chemical Composition

**Figure 1:**
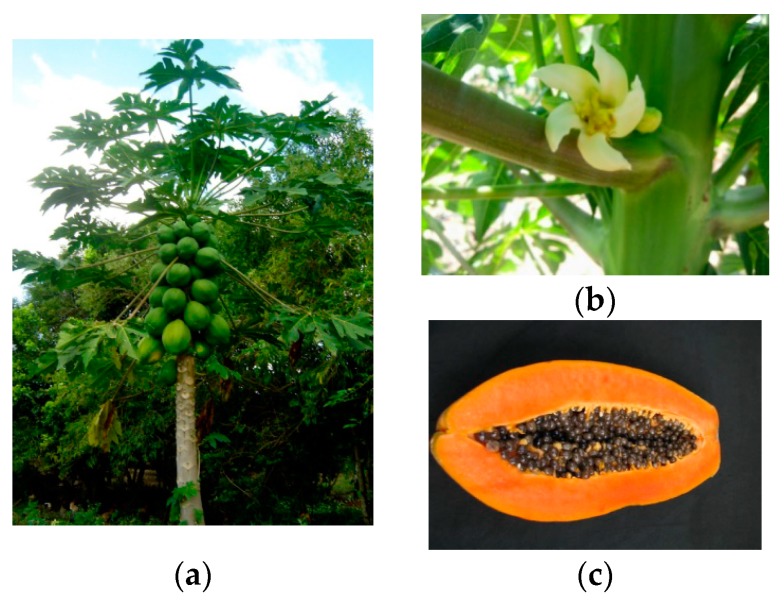
Images of *Carica papaya* L. (papaya CV Formosa): (**a**) Tree with leaves and green fruits, (**b**) female flower, and (**c**) ripe fruit with seeds and pulp. Photos: L. F. Santana.

The tree *C. papaya* is native to Central and South America and is one of the most cultivated fruit plants in the world, especially in tropical and subtropical areas [6]. It is a herbaceous perennial plant, with a milky latex that can reach 12 m in height. It has a year-round fruit production, and each fruit weighs between 1000 and 3000 g [18] (Figure 1). 

The fruit of *C. papaya* is considered one of the most common fruits in relation to human consumption and provides a favorable cost benefit in consideration of its nutritional value, with a low caloric content (Table 1) and rich concentration of vitamins and minerals (Table 2) [19].

Among the most commercialized fruits, such as apple, banana, water melon, and orange, papaya has the highest concentrations of vitamin C (61.8 mg·100 g^−1^), vitamin A (328 mg·100 g^−1^), riboflavin (0.05 mg·100 g^−1^), folate (38 mg·100 g^−1^), thiamine (0.04 mg·100 g^−1^), niacin (0.34 mg·100 g^−1^), calcium (24 mg·100 g^−1^), iron (0.1 g·100 g^−1^), potassium (257 mg·100 g^−1^), and fiber (0.8 g·100 g^−1^), as well as presenting a low caloric value (32 kcal·100 g^−1^ ripe fruit) and being one of the preferred fruits for weight loss. In addition, it has a high carotene content when compared with other fruits [5,20].

The green fruit is used in preparations, such as salads, cakes, ice creams and juice, without carotene [20], but with all of the other nutrients listed in Table 1 and Table 2. Besides the ripe papaya pulp, the consumption of other parts, such as the seeds and leaves, is appropriate, since they have a higher nutritional value and more fibers [7]. The leaves and seeds present a higher carbohydrate content, compared with the fruit pulp, presenting 78.2 g and 436 g·100 g^−1^, respectively (Table 1), and the same is observed for the values of proteins (5.8 g and 2.63 g·100 g^−1^), lipids (1.4 g and 3.1 g·100 g^−1^) and fibers (13.1 g and 2.13 g·100 g^−1^). Consequently, they have a higher caloric value (seeds with 212.7 kcal and leaves with 348.6 kcal) [18].

Compared with the seeds and pulp, the concentrations of vitamins and minerals are different in the leaves, because they play an important role in fruit development [21]. For example, in relation to minerals, the contents of magnesium, iron, potassium and calcium are higher in the leaves (the leaves have 366.1 mg and the seeds 54.4 mg·100 g^−1^). Regarding vitamins, except for C, the leaves present a higher content, with the highest concentration in the pulp, as shown in Table 2 [7,22].

### 2.2. Phytochemical Composition

Different parts of the *C. papaya* plant, such as the fruits, seeds, roots, leaves, stem and latex were found to have important bioactive compounds, which, in turn, may exert medicinal effects. The methanolic extract of unripe fruits exerted antioxidant activity in vivo, for the presence of compounds, such as quercetin and β−sitosterol [20]. Other studies detected considerable quantities of total phenols (203 mg·100 g^−1^ extract) [22] in the methanolic extract of the papaya pulp, while terpenoids, alkaloids, flavonoids and saponins were identified in the water extract [9] (Table 3). Besides, in papaya seed extracts, the presence of benzyl isothiocyanate [13] and expressive quantities of glucosinolates were observed [12].

Evaluating the oil extracted from the seeds, the main quantified fatty acid was oleic acid (71.30%), followed by palmitic (16.16%), linoleic (6.06%), and stearic acids (4.73%) (Table 3) [25]. The predominant tocopherols were α and δ-tocopherol, with 51.85 and 18.9 mg·kg^–1^, respectively. The β-cryptoxanthin (4.29 mg·kg^–1^) and β-carotene (2.76 mg·kg^–1^) were the quantified carotenoids, and the content of total phenolic compounds was 957.60 mg·kg^–1^ [26].

Studies showed that *C. papaya* leaves present tocopherol [24], lycopene [14], flavonoids [25] and benzyl isothiocyanate [23]. Another important study demonstrated that the phytochemical composition of ethanolic, methanolic, acetate and water extracts of *C. papaya* leaves is independent of the type of extract, detecting polyphenols, flavonoids, saponins and pro-anthocyanins, besides the antioxidant activity, evaluated by the method 1,1-diphenyl-2-picrylhydrazyl (DPPH). However, the water extract had superior values of polyphenols (23.1 mgGAE/g) and antioxidant activity (166 µgTE/g), while the ethanolic extract had the highest concentrations of flavonoids (17.1 mgCE/g), saponins (82.8 mgAes/g) and pro-anthocyanins (7.91 mgCE/g) [15].

## 3. Medicinal Properties of *C. papaya*

*Carica papaya* contains important nutrients (Table 1 and Table 2) and bioactive compounds, such as antioxidants, vitamins, and minerals (Table 3), with nutraceutical characteristics and potential beneficial effects on health [5]. Studies evaluated the actions of *C. papaya* in recovery from drug-induced hepatoxicity in rodents [27,28,29,30], e.g., by carbon tetrachloride (CCl_4_), considered a potent inducer of toxic effects in the liver for being highly metabolized in bodily tissues because of the high reactivity of halogenated metabolites (CCl_3_ and Cl), and such activation of metabolites liberate the active oxygen species (ROS). Another drug in question was acetaminophen (600 mg·100 g^−1^), an analgesic and anti-pyretic, which causes acute hepatocellular damage that can be lethal if not treated [27].

Among the main effects that extracts of different parts of *C. papaya* demonstrated, in recovery from toxic effects on the liver, are the decrease in hepatic damage with the increase in antioxidant enzymes such as superoxide dismutase (SOD), glutathione (GSH), and catalase in the liver and decreases in the enzymes alanine aminotransferase (ALT), aspartate aminotransferase (AST), and alkaline phosphatase (ALP) [27,28,29,30,31]. Similar data were observed in nephrotoxicity induced by CCl4 in rats treated with *C. papaya* seed water extract, depending on the dose and time of treatment. The results showed a drop in biochemical parameters, such as the serum levels of uric acid, urea, and creatinine, besides the renal protecting ion, constated by histological evaluation after recovery from renal lesions [32].

Besides the effects on hepatic and renal toxicity, *C. papaya* displayed antimicrobial [33], anti-amoebic [34], anti-parasitic [12,13], and anti-malaria actions [11]. The use of *C. papaya* leaf water extract at different concentrations (25, 50, 100, 200 mg·mL^−1^) had antimicrobial activity on the inhibition of some human pathogens, such as *Escherichia coli*, *Pseudomonas aeruginosa*, *Kleibseilla pneumoniae*, *Staphylococcus aureus,* and *Proteus mirabilis* [33]. Another study, utilizing the same type of extract at the dose of 100 mg·mL^−1^ found anti-amebic activity against *Entamoeba histolytica* [34]. Furthermore, *C. papaya* seeds had an activity on human intestinal parasites (*Caernorhabditis elegans*), without considerable side effects, owing to the presence of B-benzylisothiocyanate, a potent anti-helminthic [12]. Studies have shown the inhibitory effects on *Plasmodium falciparum* (malaria) in vitro, while the extract from the green fruit pulp of *C. papaya* demonstrated the highest anti-malaria activity, in comparison with different extracts of other tested plants [11]. 

Other studies showed the action of the water extract of *C. papaya* leaves (20 mg·mL^−1^) on proliferation inhibition in strains of solid tumor cells in trials in vitro, e.g., cervical carcinoma (Hela), breast adenocarcinoma (MCF-7), hepatocellular carcinoma (HepG2), lung adenocarcinoma (PC14), pancreatic carcinoma (Panc-1) and mesothelioma (H2452) in a dose-dependent manner, suggesting the anti-tumoral action of the extract. To determine whether the proliferation inhibition was associated with decreased cell viability, the water extract of *C. papaya* leaves was shown to inhibit proliferation responses of hematopoietic cell strains, including T-cell lymphoma (Jurkat), plasma cell leukemia (ARH77), Burkitt’s lymphoma (Raji), and large-cell anaplastic lymphoma (Karpas-299). In addition, the *C. papaya* leaf extract showed immunomodulatory activity on peripheral human blood mononuclear cells [17].

Antiulcerogenic actions were verified with the use of *C. papaya* seed water extract (50–100 mg/kg), the same action being observed using the methanolic extract, showing gastro-protective activity in animals, in both prevention and treatment models of gastric ulcer [35]. In addition, the *C. papaya* seed extract was able to reduce the contractility of rabbit jejunum smooth muscle—the responsible compound being benzyl isothiocyanate [36]. 

Effective anti-inflammatory actions were verified by applying *C. papaya* leaf ethanolic extract (25–250 mg·kg^−1^) on carrageenan-induced paw edema in rats. However, after the ulcerogenic activity tests, the extract with the highest concentration produced a mild irritation of the gastric mucosa [37]. Besides the effects on inflammation, *C. papaya* showed wound healing properties. It is known that diabetic patients often have persistent difficulty in healing and require the delicate handling of wounds, demanding appropriate care. The topical use of the water extract of green fruits of *C. papaya* on wounds in diabetic rats, induced by streptozotocin (STPZ, 50 mg·kg^−1^), exhibited a 77% reduction of the wound, induced by excision, with faster epithelization, compared with the control group, which received Vaseline [10]. Similar results in the healing of wounds induced by excision were observed on alloxan-induced diabetic rats (150 mg·kg^−1^), which received a water extract of green fruits of *C. papaya*, the healing actions being attributed to the active component, papain, which led to the enzymatic debridement of wounds, and the fruit vitamin C content, since it is essential for the conversion of proline to hydroxyproline, a specific marker and component of the granulation tissue of the extracellular matrix in wounds [38,39]. In this way, besides possessing edible and tasty fruits, different parts of *C. papaya* are characterized by the quality of nutrients and bioactive compounds with medicinal properties that may be used in traditional medicine as an alternative or adjuvant in the treatment of some pathological conditions.

## 4. Effects of *C. papaya* L. on Metabolic Syndrome 

Obesity consists of an excessive accumulation of body fat, which can represent a serious health risk and involves several ethological factors, including social, behavioral, environmental, cultural, psychological, metabolic, and genetic factors [40]. It is known that excessive fat accumulation, mostly visceral, can be an important condition in the development of metabolic dysfunctions, such as arterial hypertension, dyslipidemia and insulin resistance, and alterations conducive to the development of diabetes mellitus type 2, cardiovascular illnesses [41] and cancer [42], such as prostate [43] and colon rectal cancer [44]. Thus, the metabolic syndrome can be defined as the set of these risk factors, i.e., a cluster of metabolic disorders associated with obesity, including insulin resistance, atherogenic dyslipidemia and hypertension, which can lead to cardiovascular illnesses [45]. 

Since adipose tissue is a source of a great number of adipokines, such as the tumor necrosis factor (TNF-α), interleukin 6 (IL-6), monocyte chemoattractant protein, also known as chemokine ligand 2 (MCP-1/CCL-2), leptin, adiponectin, and resistin, among others [46], the larger the accumulation of adipose tissue, the higher the production of these adipokines. This leads to an imbalance in their secretion, with increased pro-inflammatory and decreased anti-inflammatory adipokines, stimulating the systemic and local inflammatory process, contributing to the development of insulin resistance [46]. Furthermore, the metabolic syndrome, besides being associated with the inflammatory process, is also related with the high production of reactive oxygen species (ROS) and, consequently, can induce insulin resistance [47,48], which is increasingly recognized as a key factor linking metabolic syndrome and liver steatosis; the last is associated with excessive fat accumulation in ectopic tissues, such as the liver, and increased circulating free fatty acids, which can further promote inflammation and endoplasmic reticulum stress [49].

For the treatment of obesity and its metabolic disorders which characterize the metabolic syndrome, there are various therapeutic approaches, either pharmacological or non-pharmacological treatments, and other methods, used as healing adjuvants. As such, the use of plants or fruits, reported since remote times as alternatives for the treatment and prevention of diseases, stand out in view of their high concentrations of vitamins, bioactive compounds and also lipidic composition, which reduces inflammatory markers, aggregates platelet, protects against thrombogenesis and oxidative stress, and prevents hypercholesterolemia, hypertriglyceridemia and hyperglycemia, which can be triggered by obesity [5].

Considering the presence of vitamins, bioactive compounds and lipids of biological and nutritional importance in *C. papaya*, several studies (summarized in Table 4) evinced relevant effects of this plant in the treatment of metabolic dysfunctions, associated or not associated with obesity, which can be considered an alternative therapeutic approach in the treatment of the metabolic syndrome. 

A preliminary study [50] demonstrated that the water extract of *C. papaya* seeds showed hypoglycemic and hypolipidemic activity in adult healthy male Wistar rats, without signs of acute toxicity. The groups received the water extract of *C. papaya* seeds, at concentrations of 100 mg, 200 mg and 400 mg/kg, and glibenclamide at 0.1 mg·kg^−1^ by gavage for 30 days. The treatments at all doses of the extract led to decreased serum levels of fasting glycemia, triglycerides, total cholesterol, LDL-c, and VLDL-c, with increased HDL-c levels, depending on the dose, and responses similar to the effects of the positive control group (glibenclamide). Such a relation with the extract concentration was observed in the lowered atherogenic index, compared with the group receiving distilled water (10 mL/kg/day) and glibenclamide (0.1 mg/kg/day). The phytochemical analyses of the extract revealed the presence of alkaloids, flavonoids, saponins, tannins, anthraquinones and anthocyanosides, and the monitored animals showed a decrease insugars, related to the metabolic effects.

Another study [51] evaluated the effects of the water extract of *C. papaya* leaves (200 mg/kg to body mass), given by gavage in adult healthy male New Zealand rabbits, treated for 24 weeks, resulting in reduced body weight, concomitant with lowered levels of fasting glycemia during the trial. Moreover, over the supplementation period, the extract had a hepatotoxic effect, manifesting an increase in serum values of aspartate transaminase (AST), aspartate aminotransferase (ALT), gamma-glutamine transferase (γ-GT) and total bilirubin. Therefore, further investigations will be necessary to evaluate toxicological effects of the extract, especially on the liver, in order to, standardize doses time of administration and side effects for a safety administration.

While such metabolic effects were observed in healthy animals, other studies elucidated the hypoglycemic action in an alloxan-induced diabetes model [52,53,54]. Adenowo et al. (2014) [52] investigated alloxan-induced (150 mg/kg/body mass) diabetic Wistar rats, treated with an ethanolic extract of *C. papaya* leaves (250 and 500 mg·kg^−1^) by gavage for 21 days, and verified reduced levels of glycemia, total cholesterol, triglycerides and LDL-c, together with increased HDL-c levels, resulting mainly from the dose of 250 mg/kg to body mass. Furthermore, they verified that the extract diminished the serum concentrations of urea, creatinine, ALT and AST, as well as the parameters of diabetic animals receiving metformin. The data corroborate the study of Maniyar (2011) [54], where the water extract of *C. papaya* leaf (400 mg/kg/body mass), given by gavage for 21 days, showed a reduction in the levels of glycemia, triglycerides and total cholesterol in alloxan-induced diabetic rats (120 mg/kg/body mass), confirmed by Johnson et al. (2015) [53], who tested the water extract of seeds and leaves of *C. papaya* (400 mg/kg/body mass), by gavage for 28 days in an experimental model of diabetes (alloxan 150 mg/kg to body mass), having observed diminished levels of glycemia total cholesterol, hepatic enzymes, ALT, AST, urea, and creatinine. Nevertheless, regarding glycemic metabolism and hypoglycemic action, the seed extract was superior to the leaf extract. 

Ezekwe et al. (2014) [55] applied the experimental model of alloxan-induced diabetes (120 mg/kg to body mass) in albino rats receiving a ration added to grated green *C. papaya* pulp, splitting the animals into three groups: control non-diabetic, diabetic and diabetic fed with added grated green *C. papaya* pulp for 28 days. The third group presented relevant effects on their metabolism, such as a reduction of weight, in the levels of glycosylated hemoglobin and in the lipidic profile, including low-density lipoprotein cholesterol (LDL-c), very low-density lipoprotein (VLDL-c), triglycerides and total cholesterol, and increased serum values of High-density lipoprotein cholesterol (HDL-c).

Metabolic effects were also observed in alloxan-induced (90 mg/kg/body mass) albino rats receiving the water extract of *C. papaya* root (500 mg/kg/body mass) and glibenclamide (5 mg/kg/body mass) by gavage for 21 days. The treatment with the extract showed improved parameters of glycemia already after 7 days of the trial, an improvement in the dyslipidemic parameters and recovery of hepatic tissues and renal dysfunction. The compounds identified include hexadecanoicacid, methylester, 10-octadecanoic acid, methyl ester, ergosta-5,22-dien-3-olacetate (3β, 22E), dianhydromannitol, 1,1,3,3,5,5,7,7,9,9,11,11-dodecamethylhexasiloxane, methyl-11-hexadecanoate, and octadecanoic acid. The compounds 10-octadecenoic acid, methylester, hexadecanoic acid, and methyl ester, were the phytochemicals most present in the root extract. Thus, they may have contributed to the cited metabolic effects [56].

Hypoglycemic effects were observed not only with the isolated administration of the leaf extract of *C. papaya* in alloxan-induced diabetic rats (180 mg/kg/body mass), but also combined with co-administrated reference antidiabetic drugs, such as metformin and glimepiride. The extract, the drug, or the combination drug + extract was administered daily by gavage in periods of a short and long duration, corresponding to 3 and 7 days, respectively. The concentrations of each product given to the animals were divided based on low and high doses, established in previous studies, as follows: extract low dose: 5 mg/kg and high dose: 10 mg/kg; glimepiride low dose: 0.2 mg/kg and high dose: 0.4 mg/kg; and metformin low dose: 50 mg/kg and high dose: 100 mg/kg, for 3 and 7 days. The same period of treatment was utilized for the combinations, glimepiride + extract and metformin + extract, and the dose combinations corresponded to high-high, high-low, low-high and low-low. The lowest concentration extract (5 mg/kg) reduced the glycemic level, but the highest concentration (10 mg/kg) accelerated the starting of the glimepiride activity, while the combination of all extracts with metformin diminished the glycemic levels after 24 h. Thus, the data demonstrated that the hypoglycemic activity of the *C. papaya* leaf extract was as effective as the hypoglycemic agents, metformin and glimepiride. However, the latter had a faster action onset, as the effect of the duration of application was dependent on the nature, i.e., on the activity strength, and on the dose. Besides, the interaction between the combination drug-extract was different for each group, since the action mechanisms of glimepiride differ from those of metformin [57]. 

In studies [58] on *C. papaya* on streptozotocin (STPZ)-induced diabetes, the crude ethanolic extract of *C. papaya* leaf (100 mg/kg/day), in comparison with the ethanolic extract of the leaves of *Pandanusam aryllifolius* (100 mg/kg/day) and the drug glyburide (10 mg/kg) by gavage for 6 days in albino mice with induced diabetes by STPZ (60 mg/kg/body mass), did not alter body weight. However, there was a reduction of glycemia, and the histology showed spleen cell regeneration, reduced the number of liver pyknotic nuclei and vacuoles, and recovered kidney cuboidal tissue. The phytochemical analyses indicated the presence of alkaloids, tannins, flavonoids and saponins, suggesting that these bioactive compounds are responsible for such effects.

A possible hypothesis for the metabolic actions of *C. papaya* extracts can be seen in the study by Juárez-Rojop et al. (2012) [59], utilizing the water extract of *C. papaya* leaves at three doses (0.75, 1.5 and 3 g·100 mL^−1^) in the drinking water of animals with induced diabetes by STPZ (60 mg/kg to body mass) and non-diabetic animals for a 4-week period. The results demonstrated that the extracts at 0.75 and 1.5 g·100 mL^−1^ diminished the levels of glycemia, as well as the serum levels of total cholesterol and triglycerides. The regeneration of pancreatic islets, with a preserved cell size, was demonstrated, and yet, a rupture of hepatocytes and accumulation of glycogen and lipids was prevented. Besides, it was verified that the metabolites of nitric oxide (NO) were reduced in diabetic rats. However, with the application of the extracts, the NO levels rose. It is known that hyperglycemia and hyperlipidemia are characterized by the inhibition of endothelial NO Synthase (eNOS) and, consequently, can result in the formation of reactive oxygen species (ROS) in relaxation, depending on the damaged endothelium, with a high formation of free radicals, concomitant with a low effectiveness of antioxidant enzymes, leading to an imbalance between the formation and the protection against free radicals in the organism. Thus, the metabolic actions in that study could be related to the increased antioxidant activity of the extract, exerted in diabetic animals.

Previous studies [60] evaluated the phytochemical composition of *C. papaya* leaf extracts on the basis of chloroform, n-hexane and ethanol and verified that the chloroform extract presented steroids and quinones among its main components, which led to the choosing of this extract for the screening of biological activity in STPZ-induced diabetic rats (60 mg/kg/body mass). 

Different doses of a chloroform extract (0, 31, 62, 125 mg/kg/body mass) of *C. papaya* leaf were given by gavage to diabetic and non-diabetic rats, and as the positive control group, diabetic rats were treated with insulin (5 U/kg, intraperitoneal) for 20 days. The data proved that the extract reduced the glycemic levels, the serum concentrations of triglycerides and total cholesterol and maintained the HDL-c at levels similar to those observed in non-diabetic rats. Furthermore, the concentrations of 31 and 62 mg/kg/body mass of the extract reduced the body weight and the levels of AST and ALT, without differences for the extract with the highest concentration (125/mg/kg/body mass).

Considering not just the systemic and biochemical actions, the extract was able to act on specific tissues, such as the pancreatic islets, either in diabetic rats induced by STPZ (60 mg/kg/body mass) and in vitro in cell cultures of pancreatic islets, which were found to be the actions of the chloroform extract of *C. papaya* leaves (31, 62, 125 mg/kg/body mass), applied by gavage for 20 days. The animals receiving the extract at concentrations of 31 and 62 mg/kg/body mass showed a reduction in fastening glycemia. On the other hand, the serum levels of insulin increased in non-diabetic rats receiving 62 mg/kg/body mass, compared with the non-diabetic group, without the extract. In cell cultures of pancreatic islets treated with STPZ (6 mg in 30 µL polyethylene glycol), a decrease in the liberation of the basal insulin culture with glucose (2 g/L) occurred. Besides, when added to the extract (6 mg in 30 µL polyethylene glycol) applied to cells with STPZ, more insulin liberation occurred. However, when STPZ was added simultaneously with the extracts (3, or 6, or 12 mg in 30 µL), insulin liberation was diminished in the three conditions, independently of the dose. However, when STPZ was added after 5 days of using the extracts, the insulin liberated from the pancreatic islets was superior to the cells of the control group, normal and similar to cells with the 6 mg extract, suggesting that the extracts have a protective action on pancreatic islets. The results are confirmed by an immune histochemical trial of the spleen, in which it was verified that the diameters and areas were larger in the groups treated with *C. papaya* extract, compared with the diabetic group [61].

Among the bioactive compounds of the major proportion in the chloroform extract are the steroids. In diabetes, changes occur in the structure and function of the absorption of intestinal glucose, e.g., an increase in glucose uptake that could cause postprandial hyperglycemia. Thus, the hypothesis is that the steroids hinder the hydrolysis of carbohydrates and the absorption of intestinal glucose by hydrolyzing enzymes limiting the levels of post prandial glucose [61,62,63]. 

It is known that diabetes mellitus is characterized by a deficiency in insulin secretion and by a low response of the organs in the action of insulin [64]. The compounds present in the *C. papaya* extract may be related to effects similar to those of insulin in glycemic metabolism, promoting glucose uptake in peripherical tissues or in the skeletal muscle and adipose tissues by a process of regeneration and revitalization of their main β-cells [60,65].

Another mechanism, which may be related to the effects of *C. papaya* on glycemic metabolism, may be the inhibition of important enzymes involved in the digestion of carbohydrates, such as α-amylase and α-glycosidase. Oboh et al. (2013) [66] demonstrated that the water extract of different parts of the green fruit of *C. papaya* was able to promote the inhibition of α-amylase and α-glycosidase in a dose-dependent way (0 to 2.0 mg/mL), and the combination of different parts of the green fruit, such as seeds, pulp and peel, in equal proportions had the best inhibitory effects on both enzymes. The α-amylase degrades complex carbohydrates in the diet into oligosaccharides and disaccharides, which are converted into monosaccharides by α-glycosidase. The liberated glucose is absorbed by the intestine, resulting in post prandial hyperglycemia. A higher inhibition of these enzymes thereby occurs, and the rise of post prandial glucose from a carbohydrate-rich diet will be significantly diminished, slowing the process of hydrolysis and uptake of carbohydrates [67,68]. 

Oxidative stress is also one of the mechanisms conducive to the development and progression of diabetes mellitus, since an exacerbated increase in the production of free radicals occurs simultaneously with the decreased mechanisms of antioxidant defenses, which can result in the cell damage of organelles and enzymes, increased lipidic peroxidation and, consequently, the development of insulin resistance [69,70]. In this way, *C. papaya* was also able to present antioxidant activity [68]. Different parts of the green fruits of *C. papaya* inhibited the lipidic peroxidation induced by sodium nitroprusside in rat pancreatic cells in vitro [66]. Sodium nitroprusside is an anti-hypertensive drug, which causes cytotoxicity by the liberation of cyanide and/or NO. Under conditions of oxidative stress, NO, together with other ROSs, such as the radical superoxide, lead to the formation of the radical peroxynitrite (ONOO-), which is a potent oxidant agent that can harm most cell components, such as proteins, DNA and membrane phospholipids [71,72]. Thus, the study showed that the extract of the pulp with the peel of green fruits can have a strong inhibitory effect on the production of malondialdehyde (MDA) and a greater ability of NO radical scavenging than seeds. Such effects are attributed to the phenolic compounds and alkaloids present in the pulp, seed and peel extracts of *C. papaya*, which are biocomponents with a high antioxidant action in removing free radicals, catalyzing chelating metals, activating antioxidant enzymes, reducing the radicals of α-tocopherol and inhibiting oxidases [64,73].

Corroborating the antioxidant actions of *C. papaya*, Salla et al. (2016) [74] reported on the antioxidant activity of the methanolic and hexanic extracts at concentrations of 50, 100 and 250 µg/mL of *C. papaya* seed on HepG2 cells, the cell strain of the human hepatoma, which incurred an induction of oxidative stress by the application of hydrogen peroxide (H_2_O_2_) (500µM). The activity of the antioxidant enzyme superoxide dismutase (SOD), catalase (CAT) and glutathione peroxidase (GPx) and levels of glutathione (GSH) were lower after the induction of oxidative stress by H_2_O_2_, and after the use of methanolic and hexanic extracts, the activity of SOD was restored, except with 50 µg/mL. The GSH levels increased with the concentration of 250 µg/mL methanolic extract and 100 and 250 µg/mL hexanic extract, and the CAT activity rose with the concentrations of 250 and 500 µg/mL, with GPx only at 250 µg/mL of methanolic extract. Besides, the highest concentrations of both extracts diminished cell viability, but this could be verified in higher proportion with the hexanic extract. The levels of flavonoids in the extracts were superior in the methanolic extract, compared with the hexanic, confirming that the antioxidant activity is related to the presence of these polyphenols.

Like glycemic metabolism, the bioactive compounds present in the *C. papaya* extracts can exert effects similar to insulin in the lipidic metabolism, as under normal conditions the insulin activates the lipoprotein lipase, hydrolyses triglycerides and inhibits the lipolysis process. Insulin deficiency, in turn, stimulates lipolysis in the adipose tissue, leading to hyperlipidemia and an accumulation of hepatic fat, decreasing the content of the enzyme lipoprotein lipase, which hydrolyses lipids, resulting in increased concentrations of serum triglycerides. Increased LDL-c levels occur because of the inhibition of the insulin action in the activity of the enzyme, β-hydroxy-β-methyl glutaryl CoA reductase (HMG-CoA reductase), which exerts an important role in cholesterol metabolism [53,66].

The action of the *C. papaya* extract on the enzyme, HMG-CoA reductase, is reported by Hasimun et al. (2018) [75], assessing specifically the actions in the lipidic metabolism of the ethanolic extract of *C. papaya* leaves (50, 100, 200 mg/kg/body mass) by gavage in Wistar rats, receiving 25% of D-fructose in drinking water for 21 days. The results showed an anti-hyperlipidemic activity of the extract at a dose of 200 mg/kg/body mass, leading to decreased total levels of cholesterol, triglycerides, and LDL-c and an increase in HDL-c. The mechanism involved is related to the inhibition of the enzyme, HMG-CoA reductase, activity in the liver, an enzyme with an important role in the synthesis of endogenous cholesterol, the inhibition of which is similar to the effects of drugs of the class of statins, such as simvastatin, used as a positive control. Besides, the phytochemical analyses revealed secondary metabolites, such as alkaloids, flavonoids, tannin, saponins, steroids/triterpenoids and quinones, suggesting that the flavonoids contained in the leaf extract, especially quercetin, could be the responsible for exerting the same mechanism as that of the statins in inhibiting HMG CoA reductase [75,76,77].

Similar data in the lipidic metabolism of *C. papaya* extract were observed in albino Wistar rats, fed with hyperlipidic diet. The effects on dyslipidemia were observed, testing the water extract of papaya seed (200 and 300 mg/body mass/day) by gavage for 5 weeks, which led to a significant reduction of total cholesterol, triglycerides, and LDL-c and an increase in HDL-c in hypercholesterolemic animals. However, the antilipidemic effects of different extract concentrations were not superior to the group receiving the reference drug, simvastatin (1.8 mg/bodymass/day) [78].

Besides the effects on glycemia and lipidic series, *C. papaya* showed actions on the systemic arterial hypertension (SAH) in animal models. After evaluating the inhibitory action of the extracts on the activity of the angiotensin-converting enzyme (ACE) in vitro, the methanolic extract of *C. papaya* leaves was chosen for the study by Brasil et al. (2014) [79]. The methanolic extract (100 mg/body mass/twice a day) was given by gavage in spontaneously hypertense Wistar rats (SHR) for 30 days. Like the reference drug (enalapril 10 mg/body mass/day), an ACE inhibitor, the methanolic extract inhibited plasmatic ACE activity, enhanced cardiac hypertrophy and normalized baroreflex sensibility, suggesting the efficiency of this extract as an anti-hypertensive [79]. The systemic arterial pressure is controlled by both the renin-angiotensin system (RAS) and baroreflex. The latter is an important short-term reflex, which controls the responses of the heart beats [80]. The uncontrolled activation of RAS has an important role in the development of cardiac hypertrophy, the ACE inhibitors being important treatment options, since ACE is an important component of RAS, which leads to the formation of angiotensin II, the main vasoconstrictor of RAS, and to the reduction of baroreflex sensibility for rising blood pressure and sympathetic regulation [81,82]. In that study, the extract effects on arterial pressure could result from the presence of different bioactive compounds, especially flavonoids, e.g., ferulic acid, caffeic acid, gallic acid and quercetin, with a suggested action on ACE inhibition [79]. Previous studies demonstrated the presence of quercetin, luteolin and kaempferol in apple peel extract, which acted as inhibitors of ACE activity in vitro [83,84].

In a model of arterial hypertension induced by deoxycorticosterone acetate (DOCA, 15 mg·100 g^−1^) in Wistar rats, the crude extract of *C. papaya* fruit (20 mg/kg), besides not presenting toxic effects, was able to generate a fast drop of arterial blood pressure and heart rate, compared with normotensive rats, and had a more potent anti-hypertensive action than hydralazine (200 mg/100 g, intravenous), a vasodilator and anti-hypertensive agent [85]. Earlier studies revealed a higher activity in the synthesis of catecholamines, e.g., a higher activity of tyrosine hydroxylase in the adrenal glands of DOCA-induced hypertense rats and in rats with renal hypertension [86,87]. Therefore, the capacity of the extract to depress the arterial pressure and the heart rate may be caused by the reduction of levels of catecholamines, liberated in response to the extract [88].

While the reviewed studies hitherto were not performed in specific models of metabolic syndrome or obesity, the achieved results demonstrated that *C. papaya* has a therapeutic potential for various types of metabolic dysfunctions, such as diabetes mellitus type 1, leading to alterations in both glycemic and lipidic metabolism, oxidative stress and in models of arterial hypertension. After those studies on the actions of *C. papaya* in metabolism, further investigations on this plant in models of obesity and metabolic syndrome are needed, which would facilitate the search for new therapeutic approaches and a better understanding of the mechanisms of action in the metabolic dysfunctions associated with obesity.

## 5. Conclusions

This review evaluated the nutritional and phytochemical composition of *C. papaya* as well as the effects of the use of several types of extract from different parts of the plant. *C papaya* exhibits curative properties, such as improvements in hepatotoxicity and nephrotoxicity induced by drugs, antimicrobial, antimalarial, anti-parasitic, antitumor, anti-inflammatory actions and wound healing effects. In relation to the metabolic dysfunctions, *C. papaya* displays hypoglycemic, hypolipidemic and antihypertensive potential and demonstrates increased antioxidant activity in experimental models in vivo and in vitro. Therefore, further studies including researches on diet-induced and genetic obesity models in addition to the isolation of specific substances from different parts of *C. papaya* will be important for the development of novel natural products on the treatment and prevention of obesity and metabolic disturbances.

## Figures and Tables

**Table 1:** Nutritional value of the macronutrients and fibers of *Carica papaya* L. (papaya) per 100 g of pulp of ripe fruit, seeds and leaves [5,18].

Component	Pulp	Seeds	Leaves
Proteins	0.6 g	2.6 g	5.8 g
Lipids	0.1 g	3.1 g	1.4 g
Carbohydrates	7.2 g	43.6 g	78.2 g
Fiber	0.8 g	2.1 g	13.1 g
Energy	32.1 kcal	212.7 kcal	348.6 kcal

**Table 2:** Value of the minerals and vitamins of *Carica papaya* L. (papaya) per 100 g of ripe fruit pulp, seeds and leaves [7,19].

Component	Pulp	Seeds	Leaves
Sodium	3 mg	ND	ND
Potassium	257 mg	344 mg	534 mg
Phosphorous	5 mg	241.5 mg	221.1 mg
Magnesium	10 mg	10.4 mg	32.4 mg
Iron	0.1 mg	0.2 mg	6.4 mg
Calcium	24 mg	54.4 mg	366.1 mg
Vitamin C	61.8 mg	11.7 mg	31.1 mg
Vitamin B9 (Folate)	38 mg	ND	ND
Vitamin B6	0.1 mg	ND	ND
Vitamin B3 (Niacin)	0.34 mg	0.26 mg	0.38 mg
Vitamin B2 (Riboflavin)	0.05 mg	0.05 mg	0.14 mg
Vitamin B1 (Thiamin)	0.04 mg	0.05 mg	0.43 mg
Vitamin A	328 mg	ND	ND
Betacarotene	888 IU	65.64 IU	659.5 IU

ND: not determined.

**Table 3:** Main phytochemical compounds present in *C. papaya* L. (papaya): ripe fruit pulp, seeds and leaves [14,15,19,23,24].

Phytochemical Composition		
Pulp	Seeds	Leaves
Glutathione peroxidase Glutathione transferase Glutathione reductase Catalase Glucose-6-phosphate Total phenols Terpenols Alkaloids Flavonoids Saponins	Benzyl isothiocyanate glucosinolates Fatty acids oleic, palmitic, linoleic and stearic Tocopherols (α and δ) β-cryptoxanthine β-carotene Carotenoids Phenolic compounds	Polyphenols Flavonoids Saponins Pro-anthocyanin Lycopene Tocopherol Benzyl isothiocyanate

**Table 4:** Summary of effects of the use of *Carica papaya* L. against metabolic dysfunctions.

Reference	Host	Used Part	Treatments	Main Effects
[50]	Healthy rats	Seed	Group 1: 10 mL distilled water Group 2: glibenclamide 0.1 mg/kg Group 3: water extract 100 mg Group 4: water extract 200 mg/g Group 5: water extract 400 mg/kg Duration: 30 days	-Reduced glycemia -Reduced triglycerides, total cholesterol, LDL-c, VLDL-c and increase HDL-c -Reduced atherogenic index and coronary arteries
[51]	Healthy rabbits	Leaves	Group 1: control Group 2: water extract 200 mg/kg Duration: 24 weeks	-Reduced weight -Reduced glycemia -Increased AST, ALT, γ-GT and total bilirubin
[52]	Rats, non-diabetic and diabetic (Alloxan 150 mg/kg)	Leaves	Grupo A: non-diabetic + 1 mL distilled water Grupo B: diabetic Grupo C: diabetic + 250 mg/kg ethanolic extract Group D: diabetic + 500 mg/kg ethanolic extract Group E: diabetic + 300 mg/kg metformin Duration: 21 days	-Reduced glycemia -Reduced total cholesterol, triglycerides, LDL-c and increased HDL-c especially at 250 mg/kg -Reduced urea, creatinine, ALT and AST
[53]	Rats, non-diabetic and diabetic (Alloxan 150 mg/kg)	Seeds and leaves	Grupo 1: non-diabetic Grupo 2: diabetic + 1 mL saline Grupo 3: diabetic + 400 mg/kg seed water extract Group 4: diabetic + 400 mg/kg leaf water extract Duration: 28 days	-Reduced glycemic levels -Reduced total cholesterol -Reduced ALT, AST, urea and creatinine
[54]	Rats, non-diabetic and diabetic (Alloxan 120 mg/kg)	Leaves	Group 1: non-diabetic Grupo 2: diabetic Group 3: diabetic + glibenclamide (0.1 mg/kg/day) Group 4, 5 and 6: diabetic + C. papaya water extract (100, 200 and 400 mg/kg) Duration: 21 days	-Reduced glycemia -Improved lipidic profile and C-reactive protein
[55]	Rats, non-diabetic and diabetic (Alloxan 120 mg/kg)	Green pulp	Group 1: non-diabetic + standard ration Group 2: diabetic + standard ration Group 3: diabetic + grated C. papaya Duration: 28 days	-Reduced weight -Reduced glycated hemoglobin -Reduced LDL-c, VLDL-c, triglycerides, total cholesterol and increased HDL-c
[56]	Rats, non-diabetic and diabetic (Alloxan 90 mg/kg)	Root	Group 1: non-diabetic Group 2: diabetic Group 3: diabetic + 500 mg/kg C. papaya root water extract Group 4: diabetic + 5 mg/kg glibenclamide Duration: 21 days	-Reduced glycemia after 7 days with extract -Reduced dyslipidemia -Recovery of hepatic tissues and renal dysfunction
[57]	Rats, non-diabetic and diabetic (Alloxan 180 mg/kg)	Leaves	* Combinations: comparison between low and high dose Group extract: diabetic + low dose (5 mg/kg) and diabetic high dose (10 mg/kg) Group glimepiride: diabetic + low dose (0.2 mg/kg) and high dose (0.4 mg/kg) Group metformin: diabetic + low dose (50 mg/kg) and high dose (100 mg/kg) *Combinations: glimepiride + extract = comparison between doses: high-high, high-low, low-high, low-low *Combinations: metformin + extract = comparison between doses: high-high, high-low, low-high, low-low Duration: short—3 days Long—7 days	-The low dose extract (5 mg/kg) reduced glycemic levels -The high dose extract (10 mg/kg) accelerated the starting of glimepiride activity -The combination extracts + metformin decreased glycemic levels after 24 h
[58]	Mice, diabetic and non-diabetic (streptozotocin 60 mg/kg)	Leaves	Group 1: non-diabetic + carboxy methylcellulose (5 mL/kg) Grupo 2: diabetic mice Group 3: diabetic + 10 mg/kg glyburide Group 4: diabetic + 100 mg/kg C. papaya ethanolic extract Group 5: diabetic + 100 mg/kg P. amaryllifolius ethanolic extract Duration: 6 days	-No weight change -Reduced glycemia -Regeneration of pancreatic cells -Reduced number of liver vacuoles and pyknotic nuclei -Recovery of kidney cuboid tissue
[59]	Rats, non-diabetic and diabetic	Seeds	Groups 1, 2 and 3: non-diabetic +water extract (0.75, 1.5, 3.0 g/100 mL) Groups 4, 5 and 6: diabetic + water extract (0.75, 1.5, 3.0 g/100 mL) Duration: 30 days	-Reduced glycemia -Reduced total cholesterol and triglycerides
[60]	Rats, non-diabetic and diabetic (streptozotocin 60 mg/kg)	Leaves	Groups 1, 2, 3: non-diabetic + chloroform extract (31, 62 and 125 mg/kg,) Groups 4, 5, 6: diabetic + chloroform extract (31, 62 and 125 mg/kg) Group 7: diabetic + insulin (5 U/kg) Duration: 20 days	-Reduced glycemic levels -Reduced triglycerides and total cholesterol and maintained HDL-c levels similar to those of the non-diabetic control -Reduced body mass at 31 and 62 mg extract/kg/body mass -Reduced AST and ALT levels at 31 and 62 mg extract/kg/body mass
[62]	(1) In vivo: Rats, non-diabetic and diabetic (streptozotocin 60 mg/kg) (2) In vitro: rat pancreatic islets	Leaves	Group 1: non-diabetic Group 2: non-diabetic + chloroform extract (62 mg/kg) Group 3: diabetic Group 4: diabetic + chloroform extract (31 mg/kg) Group 5: diabetic + chloroform extract (62 mg/kg) Group 6: diabetic + chloroform extract (125 mg/kg) Group 7: diabetic + insulin (5 U/kg) Duration: 20 days	-Reduced fastening glycemia at 31 and 62 mg extract/kg -Increased insulin in non-diabetic animals + 62 mg/kg compared with non-diabetic animals, without extract -Protecting action on pancreatic islets cells
[66]	In vitro: rat spleen	Parts of green fruit -Seeds -Peel -Pulp -Pulp + peel Seeds + Peel + Pulp	Cells + (0 to 2 mg/mL) water extract	-Inhibition of the enzymatic activity of α-amylase and β-glycosidase, dose dependent (0–2 mg/mL) -Greater inhibitory action of water extracts of pulp + peel on lipidic peroxidation induced by nitroprusside -Greater ability of NO radical scavenging of pulp + peel extracts, dose dependent (0 to 0.75 mg/mL)
[74]	In vitro: HepG2 cells	Fruits	Hexanic and methanolic extracts (50, 100 and 250 µg/mL)	-Increased antioxidant activity of SOD, CAT, GPx and GSH, dose dependent and dependent on extract type -1 mg/mL methanolic extract inhibited 50% DPPH (2,2-diphenyl-1-picrylhydrazyl) -Reduced cell viability, dose dependent
[75]	Rats	Leaves	Group 1: without D-fructose Group 2: added D-fructose Group 3, 4 and 5: added D-fructose + ethanolic extract (50, 100, 200 mg/kg) Duration: 21 days	-Reduced total levels of cholesterol, triglycerides, LDL-c and increased HDL-cat 200 mg/kg/ -Inhibition of HMG-CoA reductase activity in the liver
[78]	Rats	Seed	Group 1: common + saline diet Group 2: hyperlipidic diet Group 3: hyperlipidic diet + 200 mg/kg water extract Group 4: hyperlipidic diet + 300 mg/kg water extract Group 5: hyperlipidic diet + simvastatin (1.8 mg/kg/day). Duration: 5 weeks	-Reduced total cholesterol, triglycerides, and LDL-c and increased HDL-c -No differences from positive control (simvastatin 1.8 mg/kg)
[79]	Rats	Leaves	Group 1: normotensive Group 2: hypertensive (SHR) Group 3: hypertensive (SHR) + methanolic extract 100 mg/kg Group 4: hypertensive (SHR) + enalapril 10 mg/kg Duration: 30 days	-Inhibition of activity of plasmatic ACE -Improvement of cardiac hypertrophy -Normalization of baroreflex sensibility
[85]	Rats (DOCA-induced hypertension 15 mg/100 g)	Fruits	Group 1: normotensive Group 2: hypertensive + fruit juice crude extract (20 mg/kg) Group 3: hypertensive + hydralazine (200 mg/100 g)	-Anti-hypertensive action, more potent than hydralazine
